# Assessing the Data Quality Dimensions of Partial and Complete Mastectomy Cohorts in the All of Us Research Program: Cross-Sectional Study

**DOI:** 10.2196/59298

**Published:** 2025-03-11

**Authors:** Matthew Spotnitz, John Giannini, Yechiam Ostchega, Stephanie L Goff, Lakshmi Priya Anandan, Emily Clark, Tamara R Litwin, Lew Berman

**Affiliations:** 1 All of Us Research Program, National Institutes of Health, 6710B Rockledge Drive, Bethesda, MD, 20817, United States, 1 301 496-4000, 1 866-760-5947; 2 Center for Cancer Research, National Cancer Institute, National Institutes of Health, Bethesda, MD, United States; 3 Life Sciences Division, Leidos, Reston, VA, United States

**Keywords:** data quality, electronic health record, breast cancer, breast-conserving surgery, total mastectomy, modified radical mastectomy, public health informatics, cohort, assessment, women, United States, American, nonmetastatic disease, treatment, breast cancer surgery, real-world evidence, data, mastectomy, female, data quality framework, therapy

## Abstract

**Background:**

Breast cancer is prevalent among females in the United States. Nonmetastatic disease is treated by partial or complete mastectomy procedures. However, the rates of those procedures vary across practices. Generating real-world evidence on breast cancer surgery could lead to improved and consistent practices. We investigated the quality of data from the *All of Us* Research Program, which is a precision medicine initiative that collected real-world electronic health care data from different sites in the United States both retrospectively and prospectively to participant enrollment.

**Objective:**

The paper aims to determine whether *All of Us* data are fit for use in generating real-world evidence on mastectomy procedures.

**Methods:**

Our mastectomy phenotype consisted of adult female participants who had CPT4 (Current Procedural Terminology 4), *ICD-9* (*International Classification of Diseases, Ninth Revision*) procedure, or SNOMED (Systematized Nomenclature of Medicine) codes for a partial or complete mastectomy procedure that mapped to Observational Medical Outcomes Partnership Common Data Model concepts. We evaluated the phenotype with a data quality dimensions (DQD) framework that consisted of 5 elements: conformance, completeness, concordance, plausibility, and temporality. Also, we applied a previously developed DQD checklist to evaluate concept selection, internal verification, and external validation for each dimension. We compared the DQD of our cohort to a control group of adult women who did not have a mastectomy procedure. Our subgroup analysis compared partial to complete mastectomy procedure phenotypes.

**Results:**

There were 4175 female participants aged 18 years or older in the partial or complete mastectomy cohort, and 168,226 participants in the control cohort. The geospatial distribution of our cohort varied across states. For example, our cohort consisted of 835 (20%) participants from Massachusetts, but multiple other states contributed fewer than 20 participants. We compared the sociodemographic characteristics of the partial (n=2607) and complete (n=1568) mastectomy subgroups. Those groups differed in the distribution of age at procedure (*P*<.001), education (*P*=.02), and income (*P*=.03) levels, as per *χ^2^* analysis. A total of 367 (9.9%) participants in our cohort had overlapping CPT4 and SNOMED codes for a mastectomy, and 63 (1.5%) had overlapping *ICD-9* procedure and SNOMED codes. The prevalence of breast cancer–related concepts was higher in our cohort compared to the control group (*P*<.001). In both the partial and complete mastectomy subgroups, the correlations among concepts were consistent with the clinical management of breast cancer. The median time between biopsy and mastectomy was 5.5 (IQR 3.5-11.2) weeks. Although we did not have external benchmark comparisons, we were able to evaluate concept selection and internal verification for all domains.

**Conclusions:**

Our data quality framework was implemented successfully on a mastectomy phenotype. Our systematic approach identified data missingness. Moreover, the framework allowed us to differentiate breast-conserving therapy and complete mastectomy subgroups in the *All of Us* data.

## Introduction

Breast cancer is one of the most common forms of cancer in females worldwide and has a lifetime prevalence of 13%. The incidence in the United States is estimated to be greater than 297,000 women annually and increases with patient age [1,2]. In addition to patient age, breast cancer risk factors include BMI, early age of menarche, late age of menopause, family history or genetic risk, and environmental exposures [3].

Nonmetastatic breast cancer is treated surgically, and approximately 30% of patients have a complete mastectomy. An alternative to a complete mastectomy is breast-conserving therapy (BCT), which consists of breast-conserving surgery and radiation therapy [4]. In multiple randomized controlled trials, BCT has been shown to have similar long-term disease-free survival to a complete mastectomy [5-8].

A recent systematic review found that patients’ choice of surgical treatment was multifaceted. Some factors that were associated with patients choosing a mastectomy over BCT were related to tumor characteristics and pathology. Others were sociodemographic or individual belief factors, such as body image, aversion to radiation, and physician preference [9]. In a prospective study of 180 patients, surgeons’ preference was the strongest predictor of surgical treatment [10]. Accordingly, there is a need to compare a complete mastectomy to a partial mastectomy, the surgical component of BCT that encompasses lumpectomy, quadrantectomy, and other BCT-related surgical interventions.

We believe that a robust characterization of partial and complete mastectomy patients with data from the *All of Us* Research Program could generate valuable real-world evidence regarding breast cancer treatment and be used to provide evidence towards best practices for patients with the disease. The *All of Us* Research Program has electronic health records (EHRs) on more than 287,000 patients from 50 health care organizations within the United States. The program does targeted enrollment of groups that are underrepresented in biomedical research. Because the *All of Us* Research Program is one of the most comprehensive and diverse observational health care databases worldwide, those findings would represent real-world data associated with partial or complete mastectomy procedures [11].

Accordingly, to date, we are unaware of a study assessing the fitness for the use of *All of Us* and focusing on mastectomy as a treatment modality. Accordingly, the primary objective of this study is to determine whether the *All of Us* data are fit for an analysis of women who had a mastectomy.

## Methods

### Observational Medical Outcomes Partnership Common Data Model

The Observational Medical Outcomes Partnership Common Data Model (OMOP CDM) is the data standard used by the *All of Us* Research Program. The OMOP CDM consists of standardized concepts and relationships, allowing for harmonizing data from different sources. OMOP CDM concepts use codes from structured medical terminologies as the source (eg, CPT4 [Current Procedural Terminology 4], *ICD-9* [*International Classification of Diseases, Ninth Revision*], and LOINC [Logical Observation Identifiers Names and Codes]). The schema consists of standardized concept relationships across data tables [12-14].

We created partial and complete mastectomy phenotypes by manual selection. First, we selected CPT4, *ICD-9* procedure, and SNOMED (Systematized Nomenclature of Medicine) codes for those procedures manually. We chose CPT4, *ICD-9* procedure, and SNOMED source codes because these are the standards that are used by the OMOP CDM. Then, we searched ATHENA for the corresponding OMOP CDM concepts [15]. The partial and complete mastectomy OMOP CDM concept sets were the basis for our phenotype queries. Additionally, we restricted the phenotype to the earliest occurrence of a procedure and to female participants who were at least 18 years or older at the time of the procedure.

### Primary Outcomes and Variables

#### Overview

We developed a data quality dimensions (DQD) framework and evaluation matrix that was adapted from Kahn et al [16]. The framework comprises 5 mutually exclusive and parsimonious dimensions that can be operationalized and applied to a mastectomy cohort as primary outcome variables: conformance, completeness, concordance, plausibility, and temporality. A prior study applied these dimensions to a ductal carcinoma in situ cohort data quality analysis [17].

#### DQD Framework

Concomitantly, we evaluated the application of the DQD framework to a mastectomy cohort. Each framework element was evaluated with respect to concept selection, internal verification, and external validation. The overarching principles of assessing the DQD include internal characteristics, described by Kahn et al [16] as verification; comparing external benchmarks as validation; and applying descriptive, inferential, and agreement statistics and data visualization. In practice, a researcher would decide whether the data associated with their constructed cohort meets their expectations for fitness of use based on the rating matrix [18]. For the DQD analysis, we selected OMOP CDM concepts related to risk factors and the medical management of breast cancer. Specifically, we included concepts that included but were not limited to breast cancer diagnoses, breast biopsies, screening and diagnostic breast imaging, endocrine therapy, anti–human epidermal growth factor receptor 2 (anti-HER2) therapy, tyrosine kinase inhibitors, chemotherapy, radiation therapy, laboratory measurements, and genetic risk factors [19,20]. Many of our codes had been validated in a prior ductal carcinoma in situ study [17]. Furthermore, a surgical oncologist (SLG) reviewed those codes, confirmed their appropriateness, and recommended additional codes for our analysis. Representative codes are shown in the supplemental appendix (Tables S1-S8 in Multimedia Appendix 1).

### Sociodemographic Characterization and Geospatial Analysis

We characterized the geospatial distribution of the mastectomy cohort participants based on state address at the time of enrollment. Also, we characterized the mastectomy cohort, the partial and complete mastectomy subgroups, and control cohort according to sex at birth, race or ethnicity, age group, education, and income.

### Analysis

*All of Us* participants enrolled between May 6, 2017, and July 1, 2022, provided consent to participate and had the option to authorize sharing of their EHRs. Upon enrollment, participants were required to fill out a basic self-reported survey, which includes information on sociodemographic characteristics, and could also consent to have additional data submitted to the program, including data from biospecimens, genomic sequences, and wearable data. All analyses presented in this paper used the *All of Us* Controlled Tier Dataset v7, released on April 20, 2023. The source data were formatted to be compatible with OMOP CDM (version 5.3.1) [21]. Additionally, the data curation team modified some of the drug table schemas for optimal use with *All of Us* data. Accordingly, we modified our queries to maximize the capture of drug exposure data. Missing data were not included in the analysis and we did not make statistical adjustments for missingness.

All programming and statistical analyses were performed in Python (version 3.7.12) and were implemented in a Jupyter Notebook (version 6.5.4). We used chi-squared statistics to test for independent association, Spearman coefficients to measure bivariate correlations, and data visualization to explore the application of the DQD. The level of significant differences was set at *P*<.05.

### Ethical Considerations

The *All of Us* Research Program complies with multiple ethical considerations. First, it has an institutional review board (IRB) that reviews the protocol, informed consent, and other participant-facing materials for the *All of Us* Research Program. The IRB follows the regulations and guidance of the Office for Human Research Protections for all studies [22]. The *All of Us* IRB determined that the data that were used in this analysis were considered non–human subjects’ research. Second, participants are provided with information on how the program operates, reasonable expectations, and participants’ rights. Participants who agree to enroll sign consent forms [23]. Third, *All of Us* participants’ data are removed of identifiers and coded to protect their privacy before they are made available to researchers. Reidentification or recontacting of participants is prohibited, and governance mechanisms ensure protection against reidentification or recontact of participants [24]. Fourth, *All of Us* participants who give blood, saliva, or urine samples receive a one-time compensation of US $25 [25]. Otherwise, no direct compensation is provided. Fifth, this paper is not focused on imaging, and thus we have not included any images in the supplemental material. In addition, we censor counts that are less than or equal to 20 to comply with program requirements for minimizing disclosure risk. Data and code used in this study are available as a featured workspace to registered researchers of the *All of Us* Researcher Workbench [26].

## Results

### Sample

In the *All of Us* database, 249,565 participants consented to participate in the study, were at least 18 years old, and selected assigned as female at birth in the *All of Us* “Basics” self-reported questionnaire. Of those, 172,401 (69%) signed an authorization to share clinical data and had at least one data record in a participating EHR. We created a cohort of 4175 (2.4%) patients with mastectomy procedures and a control cohort of 168,226 (97.6%) female participants who did not have a mastectomy. Out of the 4175 female participants who had mastectomy procedures, 316 (7.6%) had both partial and complete mastectomy procedures. The first occurrence of the procedure code was used for subgroup assignment.

**Figure 1.**
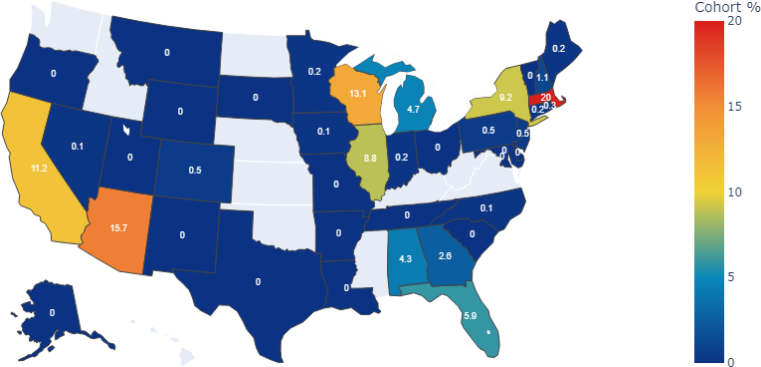
Geospatial analysis of a mastectomy cohort (partial or complete). States with a white-gray fill color contributed no participants to the cohort. Data source: The *All of Us* research program.

We plotted the mastectomy (partial or complete) cohort’s geospatial distribution to assess whether our cohort was distributed equally across the United States (Figure 1). A total of 835 (20%) participants of our cohort had medical records from Massachusetts, 656 (15.7%) from Arizona, 547 (13.1%) from Wisconsin, 468 (11.2%) from California, 386 (9.3%) from New York, 369 (8.8%) from Illinois, 245 (5.9%) from Florida, and 197 (5.9%) from Michigan. Many states had mastectomy cases for fewer than 20 participants in our cohort and were not reported due to disclosure risk guidelines.

Table 1 provides a breakdown of the cohort by sex at birth, race or ethnicity, age group, education, and income by partial (n=2607) and complete (n=1568) mastectomy. Among the participants who underwent partial or complete mastectomy, the majority were white (66.9% and 70.4%, respectively), with procedures peaking between 40 and 79 years (89.5% and 82.6%, respectively), differ in achieving college or higher degree (52.2% and 58%, respectively), and household income greater or equal to 100k (27% and 32.3%, respectively). A similar sociodemographic comparison was performed for the mastectomy cohort and the control group (Table S8 in Multimedia Appendix 1).

**Table 1.** Sociodemographic characteristics of *All of Us* partial and complete mastectomy cohorts.

Demographic category	Partial mastectomy, n (%)	Complete mastectomy, n (%)	*P* value
Assigned sex at birth			—^a^
Female	2607 (100)	1568 (100)	
Race or ethnicity^b^			.14
Asian	80 (3.1)	55 (3.5)	
Black	380 (14.6)	180 (11.5)	
Hispanic	379 (14.5)	226 (14.4)	
Middle East and North Africa, Native Hawaiians, and Pacific Islanders	30 (1.2)	n≤20	
White	1744 (66.9)	1104 (70.4)	
Prefer not to answer, or skip	47 (1.8)	28 (1.8)	
None of these	27 (1.0)	n≤20	
Age at procedure (years)			<.001
18‐39	187 (7.2)	258 (16.5)	
40‐59	1140 (43.7)	851 (54.3)	
60‐79	1193 (45.8)	444 (28.3)	
≥80 or <18	87 (3.3)	n≤20	
Education			.02
Never attended or grades 1 through 4 (primary)	21 (0.8)	n≤20	
Grades 5 through 8 (middle school)	47 (1.8)	23 (1.5)	
Grades 9 through 11 (some high school)	89 (3.4)	52 (3.3)	
Grade 12 or GED^c^ (high school graduate)	346 (13.3)	187 (11.9)	
College 1 to 3 (some college, associate’s degree, or technical school)	705 (27.0)	365 (23.3)	
College graduate	713 (27.4)	454 (29.0)	
Advanced degree (Master’s, Doctorate, etc)	647 (24.8)	456 (29.0)	
Prefer not to answer, or skip	39 (1.5)	n≤20	
Annual household income (US $)			.03
Less than 10k	199 (7.6)	89 (5.7)	
10k-25k	243 (9.3)	149 (9.5)	
25k-35k	173 (6.6)	91 (5.8)	
35k-50k	202 (7.8)	110 (7.0)	
50k-75k	289 (11.1)	168 (10.7)	
75k-100k	270 (10.4)	162 (10.3)	
100k-150k	311 (11.9)	199 (12.7)	
150k-200k	149 (5.7)	120 (7.7)	
More than 200k	246 (9.4)	186 (11.9)	
Prefer not to answer	390 (15.0)	223 (14.2)	
Skip	135 (5.2)	71 (4.5)	

a Not applicable.

b More than one race or ethnicity category could have been selected.

c GED: General Educational Development.

### Conformance

**Figure 2.**
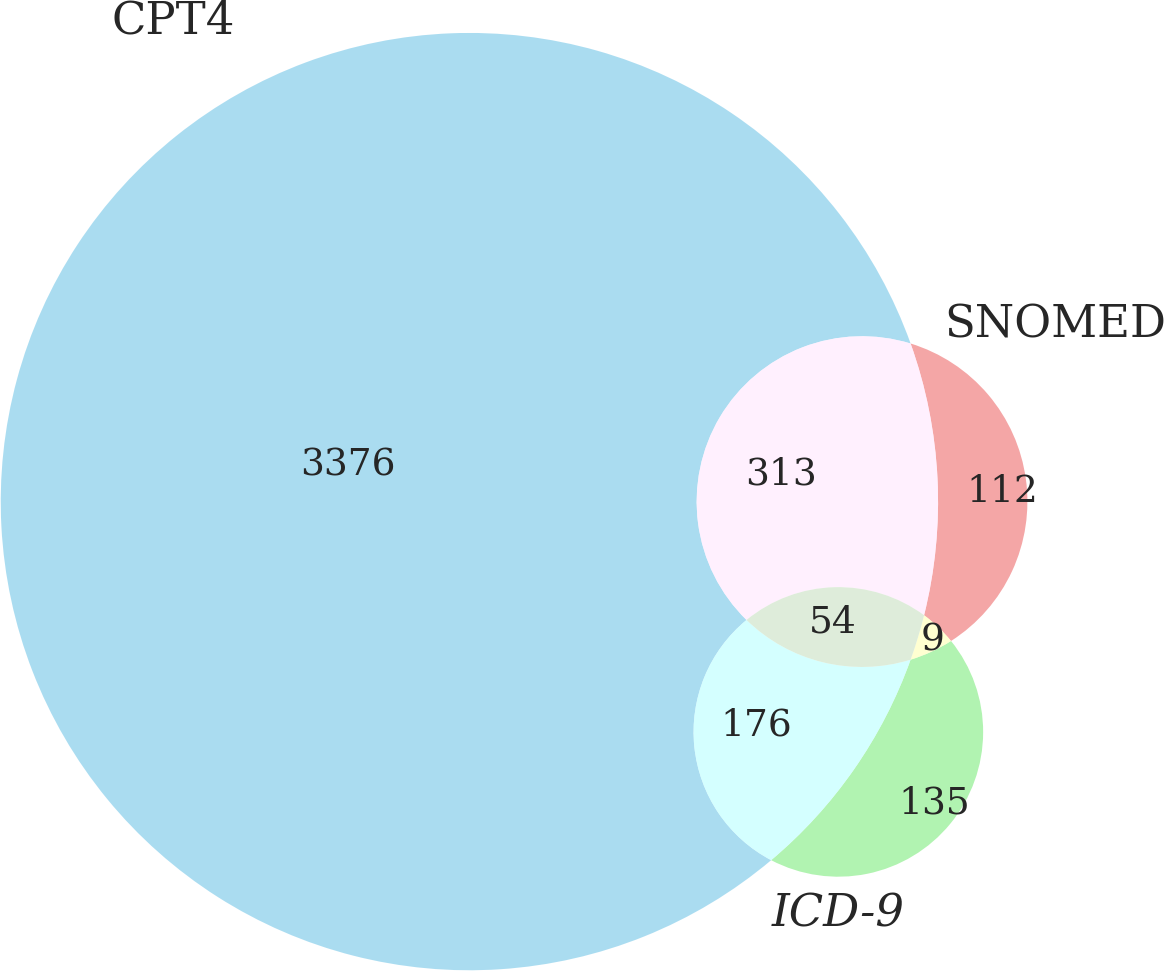
The butterfly plot of CPT4 (left), SNOMED (top right), and *ICD-9* (bottom right) mastectomy procedure codes. CPT4: Current Procedural Terminology 4; *ICD-9*: *International Classification of Diseases, Ninth Revision*; SNOMED: Systematized Nomenclature of Medicine. Data source: The *All of Us* research program.

Data elements can be assessed according to standards. The *All of Us* Program uses SNOMED as a standard vocabulary. We created a butterfly plot to determine the overlap between CPT4, *ICD-9* procedure, and SNOMED procedure codes, as shown in Figure 2. Of the 4175 female participants in our cohort, 3376 (80.9%) had CPT4 codes only, 313 (7.5%) had both CPT4 and SNOMED codes, 176 (3.2%) had CPT4 and *ICD-9* procedure codes, and 63 (1.5%) had *ICD-9* procedure and SNOMED codes. A total of 54 (1.3%) female participants had overlapping CPT4, SNOMED, and *ICD-9* procedure codes (Figure 2). Thus, the overlap among standards was low.

To characterize the source data variance in the standards, we calculated the counts of the partial or complete mastectomy CPT4 codes in our cohort (Table 2). Of the 50 EHR-contributing *All of Us* sites, 24 reported mastectomy CPT4 codes. CPT4 code 19301 (“mastectomy, partial”) was reported the most frequently by every site that contributed data to our cohort. The sets of distinct CPT4 codes that each site reported varied substantially, with the median site using 6 different CPT4 codes. We used data from within our cohort to verify conformance. However, we did not validate this dimension against an external benchmark because one was not available.

**Table 2.** Mastectomy Current Procedural Terminology 4 (CPT4) counts by code. Codes with ≤10 counts were omitted. Data source: The *All of Us* research program.

CPT4 code	Count
19301^a^	2366
19303^b^	1304
19307^c^	358
19302^d^	160
19160^e^	126
19180^f^	53
19162^g^	48
19304^h^	44
19240^i^	35

a CPT4 code 19301=mastectomy, partial (eg, lumpectomy, tylectomy, quadrantectomy, and segmentectomy).

b CPT4 code 19303=mastectomy, simple, complete.

c CPT4 code 19307=mastectomy, modified radical, including axillary lymph nodes, with or without pectoralis minor muscle, but excluding pectoralis major muscle CPT4.

d CPT4 code 19302=mastectomy, partial (eg, lumpectomy, tylectomy, quadrantectomy, segmentectomy); with axillary lymphadenectomy.

e CPT4 code 19160=mastectomy, partial.

f CPT4 code 19180=mastectomy, simple, complete.

g CPT4 code 19162=mastectomy, partial, with axillary lymphadenectomy.

h CPT4 code 19304=mastectomy, subcutaneous.

i CPT4 code 19240=mastectomy, modified radical, including axillary lymph nodes, with or without pectoralis minor muscle, but excluding pectoralis major muscle.

### Completeness

We used concept prevalence within our cohort to evaluate data completeness. Table 3 shows the counts and percentages of female participants who did and did not have partial or complete mastectomy procedures, for which each specific clinical measure and intervention was present in the *All of Us* EHR at least once. The participants in our partial or complete mastectomy cohort had a higher prevalence of breast cancer associated OMOP CDM concepts compared to female control cohort participants who did not have a partial or complete mastectomy code. Specifically, comparing females who had a partial or complete mastectomy to females who had neither showed increased prevalence of diagnostic mammography (70.7% vs 13.5%), biopsy (61.2% vs 3.3%), or endocrine therapy (51% vs 1.8%), or chemotherapy (25.1% vs 4.3%). A *χ*^2^ test indicated that partial or complete mastectomy procedures were associated with clinical measures and interventions (*P*<.001).

**Table 3.** Clinical measures and interventions for female participants who had a mastectomy and who did not have a mastectomy. Data source: The *All of Us* research program.

Clinical measure	Mastectomy cohort, n (%)	Nonmastectomy cohort, n (%)	*P* value
Procedures			<.001
Breast biopsy	2554 (61.2)	5625 (3.3)	
Diagnostic mammography	2951 (70.7)	22,731 (13.5)	
Radiation therapy	1656 (39.7)	1644 (1.0)	
Screening mammography	2143 (51.3)	45,071 (26.8)	
Surgery	4175 (100.0)	0 (0)	
Medications			<.001
Anti-HER2^a^	221 (5.3)	151 (0.1)	
CDK^b^ 4/6 inhibitors	60 (1.4)	138 (0.1)	
Chemotherapy	1046 (25.1)	7152 (4.3)	
Endocrine therapy	2130 (51.0)	3109 (1.8)	
Goserelin	106 (2.5)	91 (0.1)	
Olaparib	≤20	41 (<0.1)	
Pembrolizumab	≤20	162 (0.1)	
Tyrosine kinase inhibitor	50 (1.2)	277 (0.2)	
Conditions			<.001
Breast cancer gene mutation	435 (10.4)	903 (0.5)	
Estrogen receptor status	235 (5.7)	180 (0.1)	

a anti-HER2: anti–human epidermal growth factor receptor 2.

b CDK: cyclin-dependent kinase.

Table 4 shows the counts and percentages for the partial and complete mastectomy subgroups. Each specific clinical measure and intervention was in the *All of Us* EHR at least once. The partial mastectomy subgroup, compared to the complete mastectomy subgroup, had a greater proportion of radiation therapy (49.4% vs 23.5%), endocrine therapy (54.7% vs 44.9%), screening mammography (58.8% vs 39%), and diagnostic mammography (77.5% vs 59.3%). By contrast, the complete mastectomy group when compared to the partial mastectomy subgroup had a greater proportion of breast cancer gene (BRCA) mutations (18%vs 5.8%). A *χ*^2^ test indicated that partial and complete mastectomy subgroup categories were associated with clinical measures and interventions (*P*<.001).

**Table 4.** Clinical measures and interventions for female participants who had a partial mastectomy and who had a complete mastectomy. Data source: The *All of Us* research program.

Clinical measure	Partial mastectomy, n (%)	Complete mastectomy, n (%)	*P* value
Procedures			<.001
Breast biopsy	1728 (66.3)	826 (52.7)	
Diagnostic mammography	2021 (77.5)	930 (59.3)	
Radiation therapy	1288 (49.4)	368 (23.5)	
Screening mammography	1532 (58.8)	611 (39.0)	
Surgery	2607 (100.0)	1568 (100.0)	
Medications			<.001
Anti-HER2^a^	111 (4.3)	110 (7.0)	
CDK^b^ 4/6 inhibitors	31 (1.2)	29 (1.8)	
Chemotherapy	574 (22.0)	472 (30.1)	
Endocrine therapy	1426 (54.7)	704 (44.9)	
Goserelin	51 (2.0)	55 (3.5)	
Olaparib	≤20	≤20	
Pembrolizumab	≤20	≤20	
Tyrosine kinase inhibitor	27 (1.0)	23 (1.5)	
Conditions			<.001
Breast cancer gene mutation	152 (5.8)	283 (18.0)	
Estrogen receptor status	162 (6.2)	73 (4.7)	

a anti-HER2: anti–human epidermal growth factor receptor 2.

b CDK: cyclin-dependent kinase.

**Figure 3.**
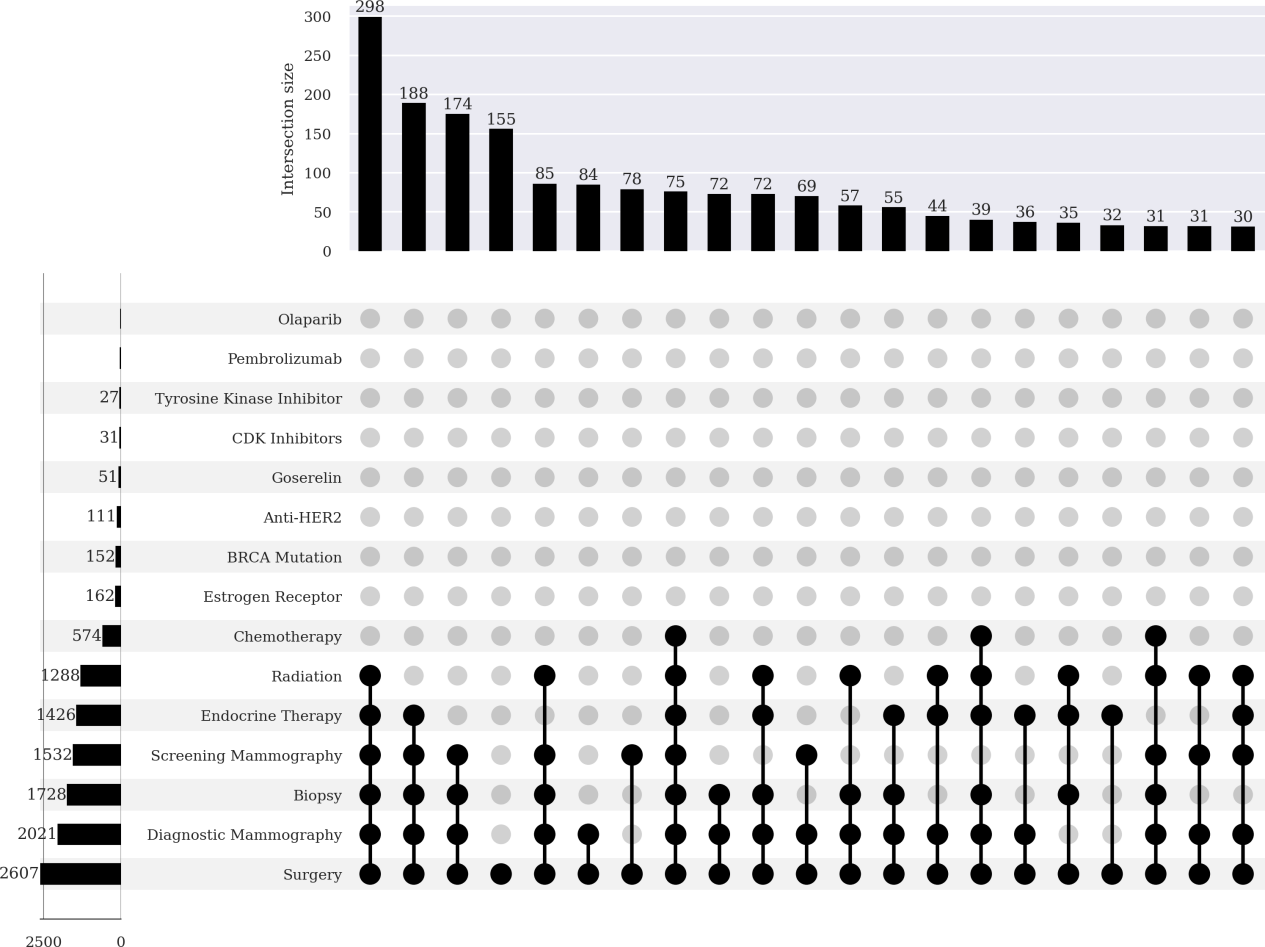
Bar chart (top) and UpSet plot (bottom) of breast cancer–related diagnosis codes, procedures, medications, and genetic tests in female participants who had a partial mastectomy. Data source: The *All of Us* research program. anti-HER2: anti–human epidermal growth factor receptor 2; BRCA: breast cancer gene; CDK: cyclin-dependent kinase.

To further characterize completeness, we used UpSet plots (Figures3  4) to assess which combinations of clinical measurements and interventions were prevalent among participants in the partial and complete mastectomy subgroups. The plots show the counts of the concept sets on the left-hand side, and the counts of concept set combinations at the top. The makeup of the combinations is indicated by the dotted lines below. The most frequent combinations in the partial mastectomy subgroup are presented in Textbox 1.

Textbox 1. **The most frequent combinations in the partial mastectomy subgroup.**

- Combination 1: Surgery, diagnostic mammography, biopsy, screening mammography, endocrine therapy, and radiation therapy (298 cases)
- Combination 2: Surgery, diagnostic mammography, biopsy, screening mammography, and endocrine therapy (188 cases)
- Combination 3: Surgery, diagnostic mammography, biopsy, and screening mammography (174 cases)

**Figure 4.**
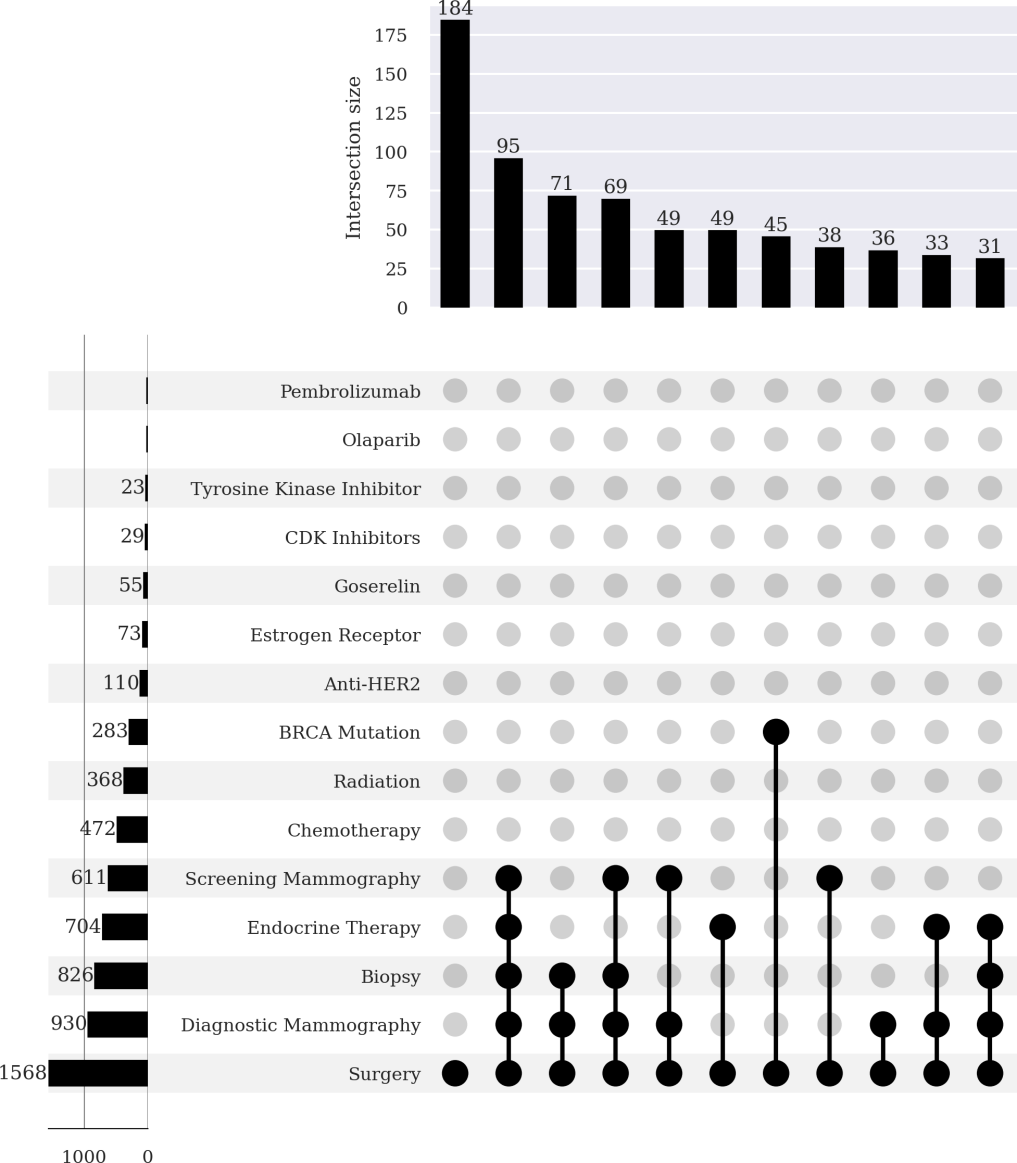
Bar chart (top) and UpSet plot (bottom) of breast cancer–related diagnosis codes, procedures, medications, and genetic tests in female participants who had a complete mastectomy. Data source: The *All of Us* research program. anti-HER2: anti–human epidermal growth factor receptor 2; BRCA: breast cancer gene; CDK: cyclin-dependent kinase.

We did not validate completeness because external benchmarks were not available.

The most frequent combinations in the complete mastectomy subgroup are presented in Textbox 2.

Textbox 2. **The most frequent combinations in the complete mastectomy subgroup.**

- Combination 1: Surgery (184 cases)
- Combination 2: Surgery, diagnostic mammography, biopsy, screening mammography, and endocrine therapy (95 cases)
- Combination 3: Surgery, diagnostic mammography, and biopsy (71 cases)

### Concordance

**Figure 5.**
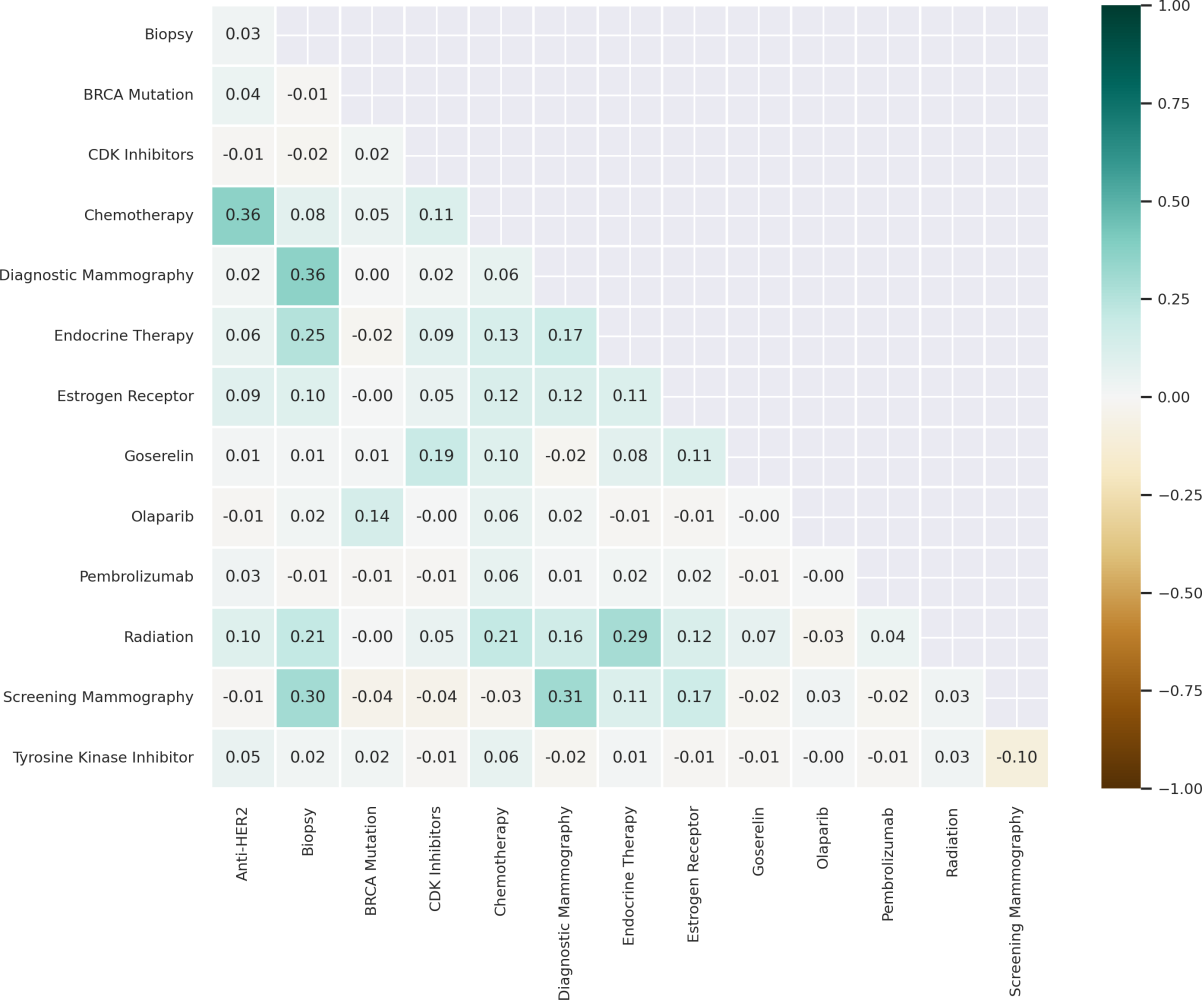
Correlogram of medications, procedures, and genetic tests in the subgroup of partial mastectomy patients. Data source: The *All of Us* research program. anti-HER2: anti–human epidermal growth factor receptor 2; BRCA: breast cancer gene; CDK: cyclin-dependent kinase.

We calculated the bivariate correlations between OMOP CDM concepts for clinical measures and interventions in the partial and complete mastectomy subgroups to measure concordance (Figures5  6). The highest bivariate correlations for the partial mastectomy subgroup were between biopsy and diagnostic mammography (*r*=0.36) and chemotherapy and anti-HER2 therapy (*r*=0.36). We also calculated the bivariate correlations for the complete mastectomy subgroup; the highest bivariate correlations were between biopsy and diagnostic mammography (*r*=0.43), radiation therapy and chemotherapy (*r*=0.38), screening mammography and diagnostic mammography (*r*=0.37), and chemotherapy and anti-HER2 therapy (*r*=0.34).

**Figure 6.**
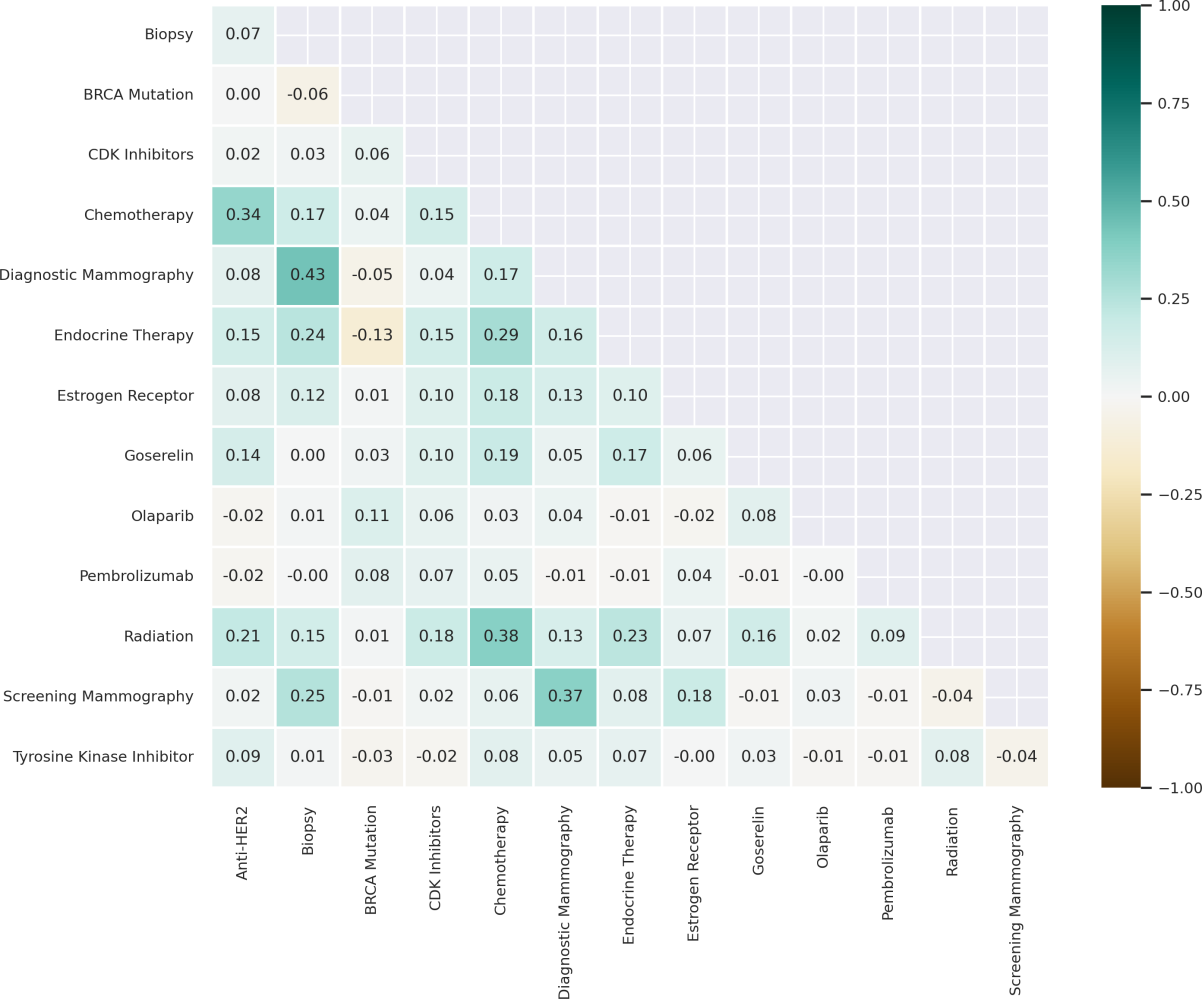
Correlogram of medications, procedures, and genetic tests in the subgroup of complete mastectomy patients. Data source: The *All of Us* research program. anti-HER2: anti–human epidermal growth factor receptor 2; BRCA: breast cancer gene; CDK: cyclin-dependent kinase.

**Figure 7.**
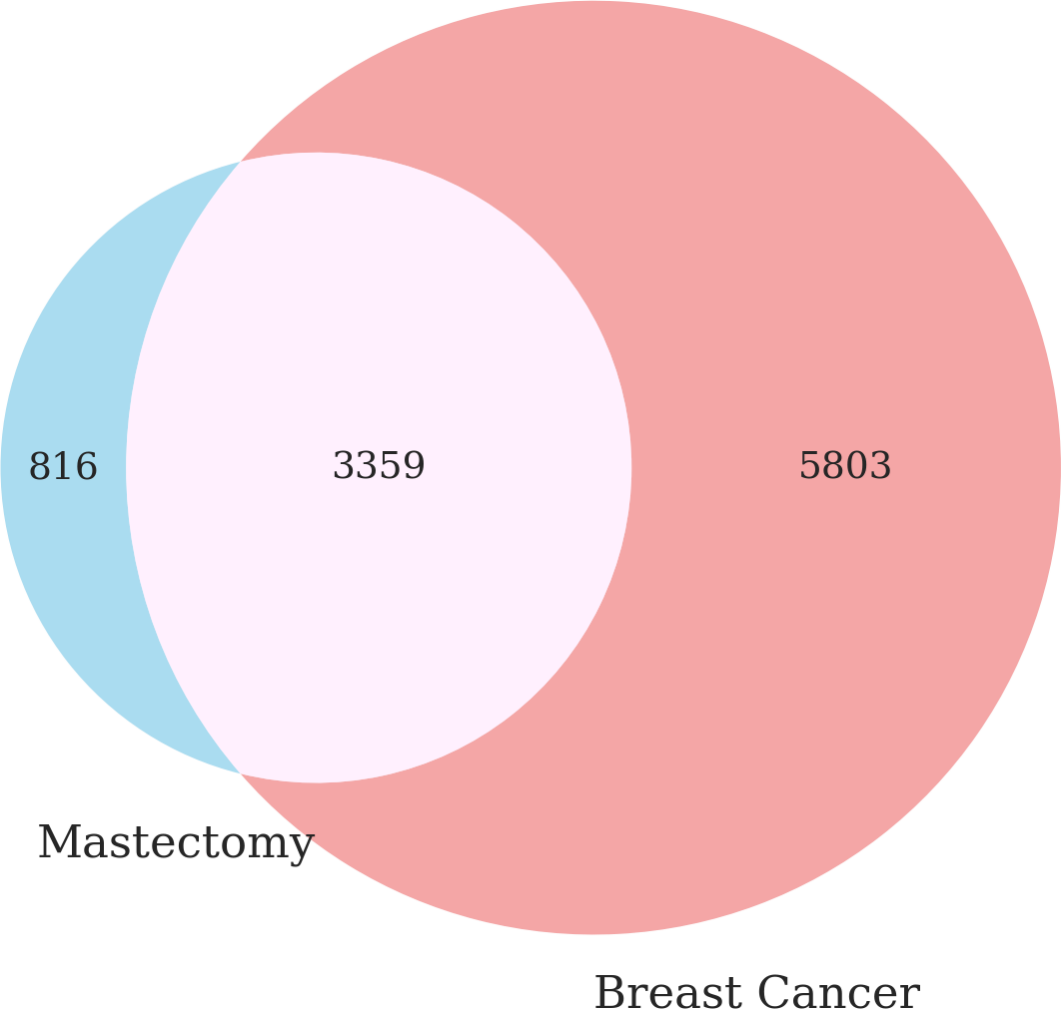
The butterfly plot of the mastectomy procedure (left) and the breast cancer diagnosis (right) codes. Data source: The *All of Us* research program.

The overlap of patients who had a mastectomy procedure and breast cancer diagnosis (eg, SNOMED 254837009 “Malignant Neoplasm of Breast”) is shown in Figure 7. Of the 816 (19.5%) of female participants who had a mastectomy code only, 277 (33.9%) had diagnosis codes for physical or radiographic findings (eg, breast lump, mammographic calcification of breast), premalignant disease, or benign disease within 1 year before the procedure.

We did not validate concordance because external benchmarks were not available.

### Plausibility

**Figure 8.**
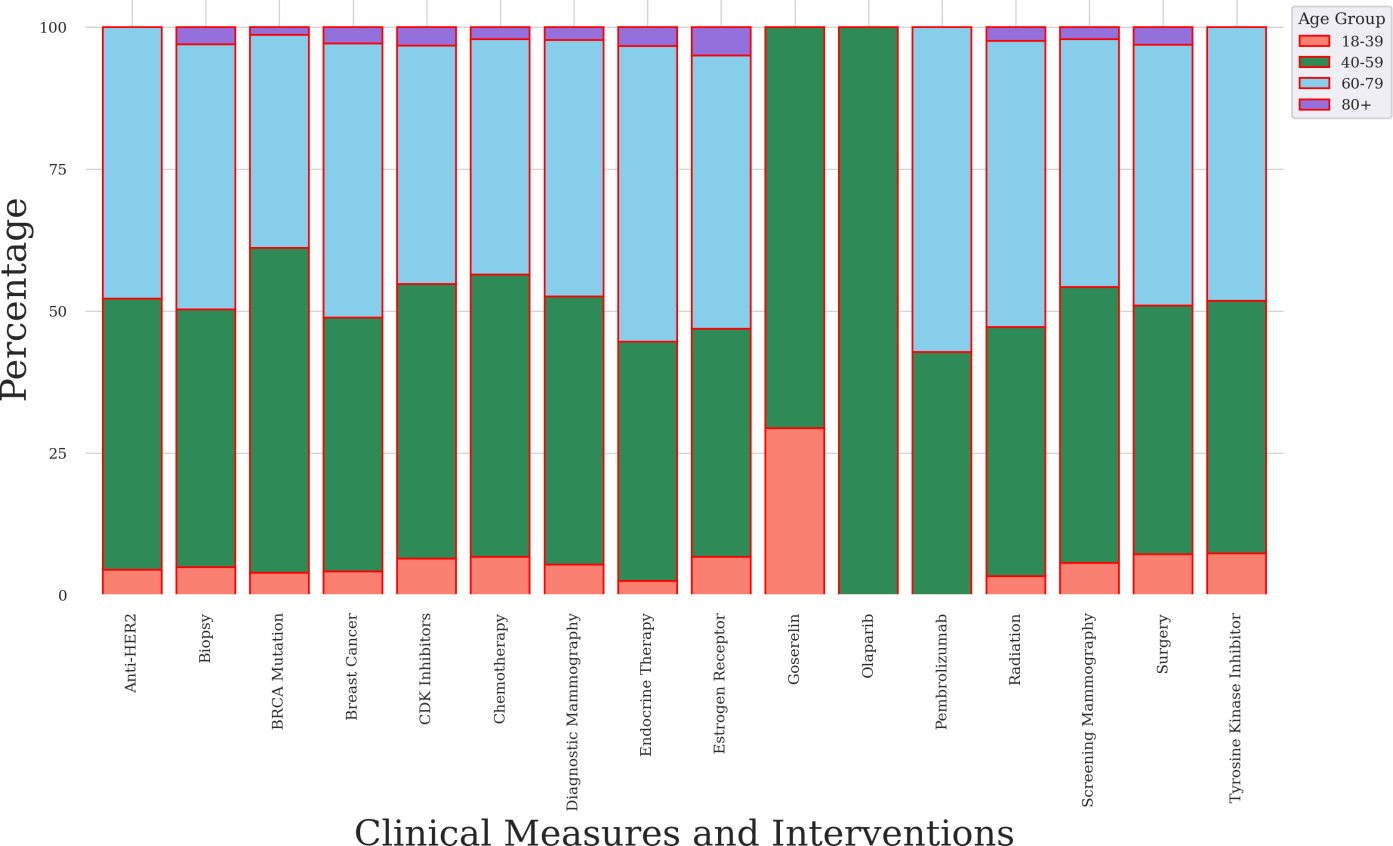
Bar chart of clinical measures and interventions for female participants who had a partial mastectomy. Data source: The *All of Us* research program. anti-HER2: anti–human epidermal growth factor receptor 2; BRCA: breast cancer gene; CDK: cyclin-dependent kinase.

We assessed plausibility by characterizing distributions of clinical measurement and intervention concepts by age group. We stratified the analysis by partial and complete mastectomy procedures (Figures8  9). We used the age at which a participant’s surgical procedure was recorded in EHR rather than other internal characteristics. Our data support a clear association between age patterns and the rate of mastectomy surgery (see Table 1) and the literature [3].

**Figure 9.**
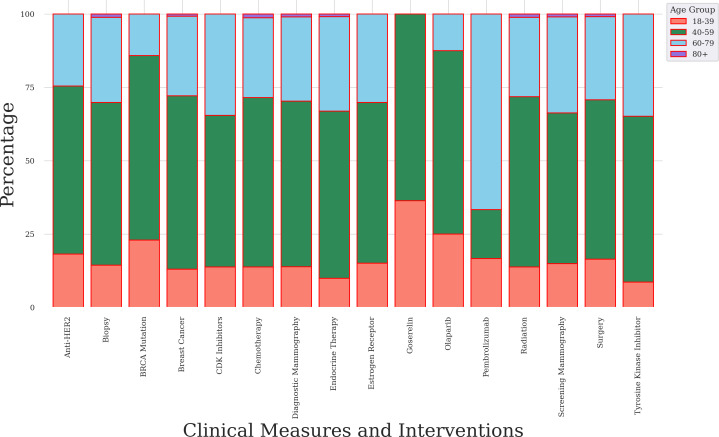
Bar chart of clinical measures and interventions for female participants who had a complete mastectomy. Data source: The *All of Us* research program. anti-HER2: anti–human epidermal growth factor receptor 2; BRCA: breast cancer gene; CDK: cyclin-dependent kinase.

For the partial mastectomy subgroup, clinical measures and interventions were most frequent in for adult female participants who were between 40 and 79 years of age (Figure 8). Specifically, BRCA mutation (57.2%) was most frequent in the 40‐ to 59-year-old group. Biopsy (46.7%), radiation therapy (50.4%), surgery (45.9%), and endocrine therapy (52%) were most frequent in the 60‐ to 79-year-old group. A *χ*^2^ test indicated that age categories were associated with clinical measures and interventions (*P*<.001).

For the complete mastectomy subgroup, the frequencies of clinical measures and interventions were highest for adult female participants who were between 40 and 79 years of age (Figure 9). Specifically, BRCA mutation (62.9%), diagnostic mammography (56.5%), biopsy (55.4%), surgery (54.3%), endocrine therapy (56.9%), radiation therapy (58%), screening mammography (51.3%), and estrogen receptor status (54.8%) concepts were most frequent in the 40‐ to 59-year-old age group. A *χ*^2^ test indicated that age categories were associated with clinical measures and interventions (*P*<.001).

We did not validate plausibility because external benchmarks were not available.

### Temporality

To assess temporality, we examined the time intervals between biopsy and mastectomy. A biopsy procedure was available for 2354 (56.4%) female participants in the partial or complete mastectomy cohort. There was a skewed time distribution from biopsy to surgery (right positive skew=9.9). Therefore, the median (5.5, IQR 3.5-11.2 weeks) better represents the distribution than the mean (18.4 weeks) for the time difference between biopsy and surgery.

We did not validate temporality because external benchmarks were not available.

## Discussion

### Principal Findings

The primary objective of this study is to determine whether the *All of Us* EHR data are fit for analyzing female participants who had a mastectomy. Indeed, this study provides valuable information to researchers on the quality of EHR data by operationalizing 5 DQD to the procedure-driven selection of the mastectomy cohort clinical measurements and interventions. We implemented concept selection and internal verification on all domains but were unable to validate them because external benchmarks were not available. Each domain provided unique information about data quality. In this study, our conformance analysis evaluated the overlap of procedure codes from different source vocabularies. The low overlap with SNOMED implies that there may be suboptimal linkage of procedure concepts with concepts from other domains because the standardized relationships may be underused. Furthermore, our method for evaluating conformance may be applicable to quantifying the amount of overlap between nonstandardized and standardized codes. The completeness DQD analysis can be used to identify disease-specific missingness in our data. The concordance analysis measures associations among concepts, which have implications for their relative missingness. The plausibility and temporality analyses are an effort to make the data quality issues transparent and comparable to existing clinical knowledge.

Despite the incompleteness of EHRs, breast cancer–related concepts were prevalent in our cohort. The correlations among those concepts were logical and consistent with the practice of treating breast cancer. For example, concepts for radiation therapy, which is an essential part of BCT, were more prevalent in the partial mastectomy subgroup. The completeness and correlations of our data allowed us to differentiate patients who had BCT from patients who had a complete mastectomy. Our cohort consisted of *All of Us* participants who had a mastectomy procedure at one of the participating sites. However, a greater number of participants may have had a mastectomy procedure at a site that was not part of our research network. Alternatively, diagnosis code-based phenotypes may have higher sensitivity and more false positives than procedure-based phenotypes.

This DQD paper is the first OMOP CDM study to evaluate the quality of partial or complete mastectomy procedure data with procedure-based phenotypes using *All of Us* EHR data. There are several distinct advantages to using a procedure-based phenotype over a diagnosis code-based phenotype. First, in the United States, procedure codes tend to be submitted by experts and can be subject to more rigorous quality checks than codes from other domains, which makes them more likely to be accurate. Second, a mastectomy is a disease-specific intervention for breast cancer. Therefore, a mastectomy phenotype should have a strong association with breast cancer. Third, procedure codes are well-defined and map to granular OMOP CDM concepts. Furthermore, the granularity of codes allows for differentiating partial from complete mastectomy procedures. Fourth, procedures are concrete events synchronizing a cohort to a point in the disease course. Synchronizing the cohort can be especially valuable for performing a treatment pathway analysis, a population-level estimation, or a patient-level prediction.

### Comparisons to Prior Work

The relative proportions of the mastectomy cohort who had partial and complete mastectomy procedures were similar to the national averages [27]. However, we found that the frequencies of multiple concepts were lower than expected in our analysis. For example, 51% of our mastectomy cohort had endocrine therapy concepts, and only 5.6% had estrogen receptor status concepts.

### Limitations

Our study had several limitations. First, the OMOP CDM breast cancer concepts had minimal information on the breast cancer stage, grade, pathology, laterality, and quadrant of a tumor. Consequently, adopting guidelines from other research networks, such as the National Comprehensive Cancer Network, was not feasible for our use because National Comprehensive Cancer Network guidelines are associated with specific tumor, node, and metastasis characteristics. Health Care Common Procedure Coding System and *International Classification of Diseases* procedure codes can help provide some information on mastectomy status; however, they are limited by their granularity and frequency in the dataset. Second, we wrote custom code to implement our phenotype and selected our concepts manually. Also, evaluating phenotypes with software packages such as CohortDiagnostics and Phevaluator is a possible future area of research. Third, our geospatial analysis was based on the participant’s location at the time of enrollment. Some participants could have had surgical treatment in another state. Because our data does not identify the site, variation in practice patterns by institution or provider was unknown. These issues are potential sources of selection bias. Notwithstanding, we recognize that institution and provider preferences can influence whether a patient undergoes a partial or complete mastectomy for breast cancer [9]. Future development with the *All of Us* Center for Linkage and Acquisition of Data may enable the effects of those preferences on patient procedure choice to be analyzed through the acquisition of health care claims data. Fourth, we restricted our analysis to female participants to reduce errors attributed to misclassification of participants’ assigned sex at birth. A study that also includes males with breast cancer, who make up 1% of the breast cancer population, would be more generalizable [28]. Fifth, there was minimal data available for an external validation comparison.

### Future Directions

Our study has shown that our data quality framework is systematic and comprehensive and can be implemented in a mastectomy use case. The results of our analysis could inform investigators about the feasibility of using *All of Us* data for follow-up studies. Furthermore, we encourage continued procedure-based phenotyping with our data. In summary, our methods can continue to assess data quality in the *All of Us* Research Program and they may lead to precision medicine studies applicable to diverse patient populations.

### Conclusions

We successfully implemented a data quality framework to evaluate whether a mastectomy phenotype that uses *All of Us* data is fit for observational health care research. Our procedure-based phenotype overcame many EHR limitations. In a subgroup analysis, we achieved reasonable differentiation of BCT from complete mastectomy patients. We encourage the continued use of procedure-based phenotypes to evaluate data quality.

## Supplementary material

10.2196/59298 Multimedia Appendix 1 Supplementary tables containing representative codes, representative medications, sociodemographic characteristics.

## Abbreviations

anti-HER2: anti–human epidermal growth factor receptor 2
BCT: breast-conserving therapy
BRCA: breast cancer gene
CPT4: Current Procedural Terminology 4
DQD: data quality dimensions
EHR: electronic health record
*ICD-9*: *International Classification of Diseases, Ninth Revision*
IRB: institutional review board
LOINC: Logical Observation Identifiers Names and Codes
OMOP CDM: Observational Medical Outcomes Partnership Common Data Model
SNOMED: Systematized Nomenclature of Medicine
