# Supplementary material for: Assessing the Data Quality Dimensions of Partial and Complete Mastectomy Cohorts in the All of Us Research Program: Cross-Sectional Study
Source: JMIR Cancer. 2025 Mar 11;11:e59298. doi: 10.2196/59298 (PMC11918980; doi:10.2196/59298)
Supplement: Multimedia Appendix 1 [file cancer-v11-e59298-s001.docx]

### Supplemental Appendix

#### Table S1: Representative Mastectomy Codes

| **OMOP Concept ID** | **OMOP Table** | **Concept Description** | **Code** | **Vocab** |
| --- | --- | --- | --- | --- |
| 42739977 | Procedure | Mastectomy, partial | 19160 | CPT4 |
| 42733288 | Procedure | Mastectomy, partial, with axillary lymphadenectomy | 19162 | CPT4 |
| 42735000 | Procedure | Mastectomy, simple, complete | 19180 | CPT4 |
| 42732332 | Procedure | Mastectomy, radical, including pectoral muscles, axillary lymph nodes | 19200 | CPT4 |
| 42733287 | Procedure | Mastectomy, radical, including pectoral muscles, axillary, and internal mammary lymph nodes | 19220 | CPT4 |
| 42735001 | Procedure | Mastectomy, modified radical, including axillary lymph nodes, with or without pectoralis minor muscle, but excluding pectoralis major muscle | 19240 | CPT4 |
| 2102733 | Procedure | Mastectomy, subcutaneous | 19304 | CPT4 |
| 2102734 | Procedure | Mastectomy, radical, including pectoral muscles, axillary lymph nodes | 19305 | CPT4 |
| 2102735 | Procedure | Mastectomy, radical, including pectoral muscles, axillary and internal mammary lymph nodes (Urban type operation) | 19306 | CPT4 |
| 2102736 | Procedure | Mastectomy, modified radical, including axillary lymph nodes, with or without pectoralis minor muscle, but excluding pectoralis major muscle | 19307 | CPT4 |
| 2102732 | Procedure | Mastectomy, simple, complete | 19303 | CPT4 |
| 2102720 | Procedure | Mastectomy, partial (e.g., lumpectomy, tylectomy, quadrantectomy, segmentectomy) | 19301 | CPT4 |
| 2102721 | Procedure | Mastectomy, partial (e.g., lumpectomy, tylectomy, quadrantectomy, segmentectomy); with axillary lymphadenectomy | 19302 | CPT4 |
| 4066543 | Procedure | Simple mastectomy | 172043006 | SNOMED |
| 42538193 | Procedure | Simple mastectomy of right breast | 741010006 | SNOMED |
| 42538192 | Procedure | Simple mastectomy of left breast | 741009001 | SNOMED |
| 4176608 | Procedure | Mastectomy of left breast | 428571003 | SNOMED |
| 4180139 | Procedure | Mastectomy of right breast | 429400009 | SNOMED |
| 4101683 | Procedure | Excision of bilateral breasts | 27865001 | SNOMED |
| 4329559 | Procedure | Simple mastectomy of bilateral breasts | 22418005 | SNOMED |
| 4182056 | Procedure | Extended simple mastectomy of bilateral breasts | 52314009 | SNOMED |
| 4033546 | Procedure | Subcutaneous mammectomy of bilateral breasts | 14693006 | SNOMED |
| 37111482 | Procedure | Subcutaneous mastectomy of left breast | 726435001 | SNOMED |
| 37111481 | Procedure | Subcutaneous mastectomy of right breast | 726434002 | SNOMED |
| 4245519 | Procedure | Subcutaneous mammectomy of bilateral breasts with synchronous implants | 60633004 | SNOMED |
| 4124154 | Procedure | Subcutaneous mastectomy and prosthetic implant | 287653007 | SNOMED |
| 42538197 | Procedure | Subcutaneous mastectomy of left breast with prosthetic implant | 741018004 | SNOMED |
| 42538198 | Procedure | Subcutaneous mastectomy of right breast with prosthetic implant | 741019007 | SNOMED |
| 43021873 | Procedure | Bilateral extended radical mastectomy | 456903003 | SNOMED |
| 3656001 | Procedure | Bilateral mastectomy for female to male transsexual | 870629001 | SNOMED |
| 4033218 | Procedure | Bilateral mastectomy with excision of bilateral regional lymph nodes | 14714006 | SNOMED |
| 4322169 | Procedure | Subcutaneous mastectomy | 70183006 | SNOMED |
| 4297155 | Procedure | Bilateral radical mastectomy | 76468001 | SNOMED |

#### Table S2: Representative Breast Cancer Medications

| **OMOP Concept ID** | **OMOP Table** | **Concept Description** | **Code** | **Vocab** |
| --- | --- | --- | --- | --- |
| *Endocrine Therapy* | | | | |
| 1436678 | Drug Exposure | Tamoxifen-containing product | 10324 | RxNorm |
| 1342346 | Drug Exposure | Toremifene | 38409 | RxNorm |
| 1348265 | Drug Exposure | Anastrozole 1 MG Oral Tablet | 84857 | RxNorm |
| 1398399 | Drug Exposure | Exemestane 25 MG Oral Tablet | 258494 | RxNorm |
| 1315946 | Drug Exposure | Letrozole | 72965 | RxNorm |
| *CDK4/6 Inhibitors* | | | | |
| 792649 | Drug Exposure | Abemaciclib | 1946825 | RxNorm |
| 45892075 | Drug Exposure | Palbociclib | 1601374 | RxNorm |
| 1592911 | Drug Exposure | Ribociclib | 1873916 | RxNorm |
| *Tyrosine Kinase Inhibitors* | | | | |
| 1359548 | Drug Exposure | lapatinib | 480167 | RxNorm |
| 793846 | Drug Exposure | neratinib | 1940643 | RxNorm |
| *Anti-Her2* | | | | |
| 42801287 | Drug Exposure | Pertuzumab | 1298944 | RxNorm |
| 1387104 | Drug Exposure | Trastuzumab | 224905 | RxNorm |
| *Chemotherapy* | | | | |
| 19052052 | Drug Exposure | Adriamycin | 42512 | RxNorm |
| 1310317 | Drug Exposure | Cyclophosphamide | 3002 | RxNorm |
| 1378382 | Drug Exposure | Paclitaxel | 56946 | RxNorm |
| 1315942 | Drug Exposure | Docetaxel | 72962 | RxNorm |
| 1344905 | Drug Exposure | Carboplatin | 40048 | RxNorm |
| 1305058 | Drug Exposure | Methotrexate | 6851 | RxNorm |
| 955632 | Drug Exposure | Fluorouracil | 4492 | RxNorm |
| 1337620 | Drug Exposure | capecitabine | 194000 | RxNorm |
| 1344354 | Drug Exposure | epirubicin | 3995 | RxNorm |
| 1338512 | Drug Exposure | doxorubicin | 3639 | RxNorm |
| 902726 | Drug Exposure | ado-trastuzumab emtansine | OMOP5042944 | RxNorm |
| *Other Category* | | | | |
| 45892579 | Drug Exposure | Olaparib | 1597582 | RxNorm |
| 45775965 | Drug Exposure | Pembrolizumab | 1547545 | RxNorm |
| 1366310 | Drug Exposure | Goserelin | 50610 | RxNorm |

#### Table S3: Representative Screening Mammography Codes

| **OMOP Concept ID** | **OMOP Table** | **Concept Description** | **Code** | **Vocab** |
| --- | --- | --- | --- | --- |
| 4077697 | Measurement | Screening mammography | 24623002 | SNOMED |
| 36713187 | Measurement | Screening mammography of bilateral breasts | 384151000119104 | SNOMED |
| 36713192 | Measurement | Screening mammography of left breast | 392531000119105 | SNOMED |
| 36713191 | Measurement | Screening mammography of right breast | 392521000119107 | SNOMED |
| 42627987 | Procedure | Screening mammography, bilateral (2-view study of each breast), including computer-aided detection (CAD) when performed | 77067 | CPT-4 |
| 4324693 | Procedure | Mammography | 71651007 | SNOMED |
| 37109442 | Procedure | Mammography of left breast | 572701000119102 | SNOMED |
| 37118087 | Procedure | Mammography of right breast | 566571000119105 | SNOMED |
| 42872575 | Procedure | Digital breast tomosynthesis | 450566007 | SNOMED |
| 37109863 | Procedure | Digital tomosynthesis of left breast | 723779007 | SNOMED |
| 37109862 | Procedure | Digital tomosynthesis of right breast | 723778004 | SNOMED |
| 37109864 | Procedure | Digital tomosynthesis of bilateral breasts | 723780005 | SNOMED |
| 4264327 | Procedure | Unilateral mammography | 6192003 | SNOMED |

#### Table S4: Representative Diagnostic Mammography Codes

| **OMOP Concept ID** | **OMOP Table** | **Concept Description** | **Code** | **Vocab** |
| --- | --- | --- | --- | --- |
| 42627940 | Procedure | Diagnostic mammography, including computer-aided detection (CAD) when performed; unilateral | 77065 | CPT-4 |
| 42628028 | Procedure | Diagnostic mammography, including computer-aided detection (CAD) when performed; bilateral | 77066 | CPT-4 |
| 42740537 | Procedure | Computer Aided detection Diagnostic Mammogram | 76082 (Deprecated) | CPT-4 |
| 2211808 | Procedure | Breast, Mammography | 77051 (Deprecated) | CPT-4 |
| 4264054 | Procedure | Ultrasonography of breast | 47079000 | SNOMED |
| 42535066 | Procedure | Ultrasonography of bilateral breasts | 1571000087109 | SNOMED |
| 42538970 | Procedure | Ultrasonography of left breast | 951000087106 | SNOMED |
| 42538971 | Procedure | Ultrasonography of right breast | 961000087109 | SNOMED |

#### Table S5: Representative Biopsy Codes

| **OMOP Concept ID** | **OMOP Table** | **Concept Description** | **Code** | **Vocab** |
| --- | --- | --- | --- | --- |
| 2102672 | Procedure | Biopsy of breast; percutaneous, needle core, not using imaging guidance | 19100 | CPT-4 |
| 44816376 | Procedure | Biopsy of breast with placement of breast localization device(s) | 19083 | CPT-4 |
| 44816374 | Procedure | Biopsy of breast with placement of breast localization device(s) | 19081 | CPT-4 |
| 2102715 | Procedure | Image guided placement, metallic localization clip, percutaneous, during breast biopsy (List separately in addition to code for primary procedure) (Deprecated) | 19295 | CPT-4 |
| 2102675 | Procedure | Biopsy of breast; percutaneous, automated vacuum assisted or rotating biopsy device, using imaging guidance (Deprecated) | 19103 | CPT-4 |
| 2102674 | Procedure | Biopsy of breast; percutaneous, automated vacuum assisted or rotating biopsy device, using imaging guidance (Deprecated) | 19102 | CPT-4 |
| 2211806 | Procedure | Stereotactic localization guidance for breast biopsy or needle placement (e.g., for wire localization or for injection), each lesion, radiological supervision and interpretation (Deprecated) | 77031 | CPT-4 |
| 44816377 | Procedure | Biopsy of breast with placement of breast localization device(s) | 19084 | CPT-4 |
| 4047494 | Procedure | Biopsy of breast | 122548005 | SNOMED |
| 4028790 | Procedure | Percutaneous needle biopsy of breast | 237376002 | SNOMED |
| 4164491 | Procedure | Surgical biopsy of breast | 274331003 | SNOMED |
| 42539496 | Procedure | Open biopsy of breast | 736615002 | SNOMED |
| 37109988 | Procedure | Ultrasonography guided biopsy of breast | 723990008 | SNOMED |
| 4302348 | Procedure | US scan and biopsy of breast | C1628493 | SNOMEDCT_US |
| 4331230 | Procedure | Vacuum assisted biopsy of breast using ultrasound guidance | 432157003 | SNOMED |
| 4028789 | Procedure | Excisional biopsy of breast | 237372000 | SNOMED |
| 4195312 | Procedure | Core needle biopsy of breast | 44578009 | SNOMED |
| 4129191 | Procedure | Breast biopsy and related procedures | 237375003 | SNOMED |
| 4172166 | Procedure | Excisional biopsy of breast mass | 42125001 | SNOMED |
| 4196880 | Procedure | Core needle biopsy of breast using ultrasound guidance | 432550005 | SNOMED |
| 37109988 | Procedure | Ultrasonography guided biopsy of breast | 723990008 | SNOMED |

#### Table S6: Representative BRCA Diagnosis Codes

| **OMOP Concept ID** | **OMOP Table** | **Concept Description** | **Code** | **Vocab** |
| --- | --- | --- | --- | --- |
| 4135411 | Condition | BRCA2 gene mutation detected | 412738007 | SNOMED |
| 4133516 | Condition | BRCA2 gene mutation not detected | 412739004 | SNOMED |
| 4135410 | Condition | BRCA1 gene mutation detected | 412734009 | SNOMED |
| 4136450 | Condition | BRCA1 gene mutation not detected | 412736006 | SNOMED |
| 40483293 | Condition | Breast cancer genetic marker of susceptibility positive (finding) | 445333001 | SNOMED |
| 45600309 | Condition | Genetic susceptibility to malignant neoplasm of ovary | Z15.02 | ICD-10-CM |
| 45600308 | Condition | Genetic susceptibility to malignant neoplasm of breast | Z15.01 | ICD-10-CM |
| 44825071 | Condition | Genetic susceptibility to malignant neoplasm of breast | V84.01 | ICD-9-CM |
| 40482469 | Condition | Breast cancer genetic marker of susceptibility not detected | 445180002 | SNOMED |

#### Table S7: Representative Radiation Therapy Codes.

| **OMOP Concept ID** | **OMOP Table** | **Concept Description** | **Code** | **Vocab** |
| --- | --- | --- | --- | --- |
| 4029715 | Procedure | Radiation oncology AND/OR radiotherapy | 108290001 | SNOMED |
| 4205728 | Procedure | Radiation therapy procedure or service | 84755001 | SNOMED |
| 44828568 | Procedure | Encounter for other and unspecified procedures and aftercare | V58 | ICD-9-CM |
| 2102718 | Procedure | Placement of radiotherapy afterloading expandable catheter (single or multichannel) into the breast | 19298 | CPT-4 |
| 2102717 | Procedure | Placement of radiotherapy afterloading expandable catheter (single or multichannel) into the breast | 19297 | CPT-4 |
| 2102716 | Procedure | Placement of radiotherapy afterloading expandable catheter (single or multichannel) into the breast | 19296 | CPT-4 |
| 2211876 | Procedure | Radiation treatment management, 5 treatments | 77427 | CPT-4 |
| 2211877 | Procedure | Radiation therapy management with complete course of therapy consisting of 1 or 2 fractions only | 77431 | CPT-4 |
| 2211878 | Procedure | Stereotactic radiation treatment management of cranial lesion(s) (complete course of treatment consisting of 1 session) | 77432 | CPT-4 |
| 2792443 | Procedure | Radiation Therapy, Breast, Beam Radiation | DM0 | ICD10PCS |
| 2859172 | Procedure | Radiation Therapy @ Respiratory System @ Beam Radiation @ Chest Wall | DB07 | ICD10PCS |
| 2791569 | Procedure | Beam Radiation of Axillary Lymphatics using Electrons, Intraoperative | D7043Z0 | ICD10PCS |
| 2791567 | Procedure | Beam Radiation of Axillary Lymphatics using Photons 1 - 10 MeV | D7041ZZ | ICD10PCS |
| 2885101 | Procedure | Radiation Therapy @ Lymphatic and Hematologic System @ Beam Radiation @ Lymphatics, Axillary | D704 | ICD10PCS |
| 2808169 | Procedure | Radiation Therapy @ Lymphatic and Hematologic System @ Beam Radiation @ Lymphatics, Axillary @ Electrons | D7043 | ICD10PCS |
| 2845891 | Procedure | Radiation Therapy @ Lymphatic and Hematologic System @ Beam Radiation @ Lymphatics, Axillary @ Electrons @ None | D7043Z | ICD10PCS |
| 2808170 | Procedure | Radiation Therapy @ Lymphatic and Hematologic System @ Beam Radiation @ Lymphatics, Axillary @ Heavy Particles (Protons,Ions) | D7044 | ICD10PCS |
| 2892952 | Procedure | Radiation Therapy @ Lymphatic and Hematologic System @ Beam Radiation @ Lymphatics, Axillary @ Photons 1 - 10 MeV | D7041 | ICD10PCS |
| 2835987 | Procedure | Radiation Therapy @ Lymphatic and Hematologic System @ Beam Radiation @ Lymphatics, Axillary @ Heavy Particles (Protons,Ions) @ None | D7044Z | ICD10PCS |
| 2812901 | Procedure | Radiation Therapy @ Lymphatic and Hematologic System @ Beam Radiation @ Lymphatics, Axillary @ Photons 1 - 10 MeV @ None | D7041Z | ICD10PCS |
| 2791570 | Procedure | Beam Radiation of Axillary Lymphatics using Electrons, Intraoperative | D7043ZZ | ICD10PCS |
| 2900531 | Procedure | Radiation Therapy @ Respiratory System @ Beam Radiation @ Chest Wall @ Electrons | DB073 | ICD10PCS |
| 2885620 | Procedure | Radiation Therapy @ Respiratory System @ Beam Radiation @ Chest Wall @ Electrons @ None | DB073Z | ICD10PCS |
| 2836306 | Procedure | Radiation Therapy @ Respiratory System @ Beam Radiation @ Chest Wall @ Heavy Particles (Protons,Ions) | DB074 | ICD10PCS |
| 2840875 | Procedure | Radiation Therapy @ Respiratory System @ Beam Radiation @ Chest Wall @ Photons 1 - 10 MeV | DB071 | ICD10PCS |
| 2789768 | Procedure | Beam Radiation of Chest Wall using Electrons | DB073ZZ | ICD10PCS |
| 2789767 | Procedure | Beam Radiation of Chest Wall using Electrons, Intraoperative | DB073Z0 | ICD10PCS |
| 2789765 | Procedure | Beam Radiation of Chest Wall using Photons 1 - 10 MeV | DB073ZZ | ICD10PCS |
| 2859321 | Procedure | Radiation Therapy @ Breast (Procedure) | DM | ICD10PCS |
| 2836626 | Procedure | Radiation Therapy @ Breast @ Beam Radiation @ Breast, Left | DM00 | ICD10PCS |
| 2836627 | Procedure | Radiation Therapy @ Breast @ Beam Radiation @ Breast, Right | DM01 | ICD10PCS |
| 2885766 | Procedure | Radiation Therapy @ Breast @ Beam Radiation @ Breast, Left @ Electrons | DM003 | ICD10PCS |
| 2859322 | Procedure | Radiation Therapy @ Breast @ Beam Radiation @ Breast, Right @ Electrons | DM013 | ICD10PCS |
| 2845733 | Procedure | Radiation Therapy @ Breast @ Beam Radiation @ Breast, Left @ Photons 1 - 10 MeV | DM001 | ICD10PCS |
| 2823271 | Procedure | Radiation Therapy @ Breast @ Beam Radiation @ Breast, Right @ Photons 1 - 10 MeV | DM011 | ICD10PCS |
| 2845734 | Procedure | Radiation Therapy @ Breast @ Beam Radiation @ Breast, Left @ Heavy Particles (Protons,Ions) @ None | DM0042 | ICD10PCS |
| 2845736 | Procedure | Radiation Therapy @ Breast @ Beam Radiation @ Breast, Right @ Heavy Particles (Protons,Ions) @ None | DM014Z | ICD10PCS |
| 2872184 | Procedure | Radiation Therapy @ Breast @ Beam Radiation @ Breast, Left @ Photons 1 - 10 MeV @ None | DM001Z | ICD10PCS |
| 2823272 | Procedure | Radiation Therapy @ Breast @ Beam Radiation @ Breast, Right @ Photons 1 - 10 MeV @ None | DM011Z | ICD10PCS |
| 2792448 | Procedure | Beam Radiation of Left Breast using Electrons | DM003ZZ | ICD10PCS |
| 2792456 | Procedure | Beam Radiation of Right Breast using Electrons | DM013ZZ | ICD10PCS |
| 2792445 | Procedure | Beam Radiation of Left Breast using Photons 1 - 10 MeV | DM001ZZ | ICD10PCS |
| 2792453 | Procedure | Beam Radiation of Right Breast using Photons 1 - 10 MeV | DM011ZZ | ICD10PCS |
| 2792449 | Procedure | Beam Radiation of Left Breast using Heavy Particles (Protons,Ions) | DM004ZZ | ICD10PCS |
| 2792457 | Procedure | Beam Radiation of Right Breast using Heavy Particles (Protons,Ions) | DM014ZZ | ICD10PCS |

#### Table S8: Sociodemographic Characteristics of All of Us Mastectomy Case and Control Cohorts. GED, General Educational Development; K, $100,000. Note: More than one race/ethnicity category could have been selected.

| Demographic Category | Mastectomy Cases N(%) | Mastectomy Controls N(%) | *P-value* |
| --- | --- | --- | --- |
|  |  |  |  |
| **Assigned Sex at Birth** |  |  |  |
| Female | 4,175 (100) | 168,226 (100) |  |
| **Race / Ethnicity** |  |  | <0.001 |
| Prefer Not To Answer | 22 (0.5) | 962 (0.6) |  |
| Skip | 53 (1.3) | 1,760 (1.0) |  |
| Asian | 135 (3.2) | 6,068 (3.6) |  |
| Black | 560 (13.4) | 34,812 (20.7) |  |
| Hispanic | 605 (14.5) | 35,661 (21.2) |  |
| Middle East and North Africa, Native Hawaiians, and Pacific Islanders | 46 (1.1) | 2,037 (1.2) |  |
| None of These | 41 (1.0) | 1,689 (1.0) |  |
| White | 2,848 (68.2) | 92,739 (55.1) |  |
| **Education** |  |  | <0.001 |
| Never Attended or Grades 1 Through 4 (Primary) | 35 (0.8) | 1,948 (1.2) |  |
| Grades 5 Through 8 (Middle School) | 70 (1.7) | 4,214 (2.5) |  |
| Grades 9 Through 11 (Some High School) | 141 (3.4) | 10,009 (5.9) |  |
| Grade 12 Or GED (High School Graduate) | 533 (12.8) | 31,586 (18.8) |  |
| College 1 to 3 (Some College, Associate’s Degree, or Technical School) | 1,070 (25.6) | 45,810 (27.2) |  |
| College Graduate | 1,167 (28.0) | 38,471 (22.9) |  |
| Advanced Degree (Master’s Doctorate, etc.) | 1,103 (26.4) | 33,069 (19.7) |  |
| Prefer Not To Answer | 23 (0.6) | 953 (0.6) |  |
| Skip | 33 (0.8) | 2,166 (1.3) |  |
| **Annual Household Income** |  |  | <0.001 |
| Less than 10k | 288 (6.9) | 22,754 (13.5) |  |
| 10k to 25k | 392 (9.4) | 20,599 (12.2) |  |
| 25k to 35k | 264 (6.3) | 12,977 (7.7) |  |
| 35k to 50k | 312 (7.5) | 13,996 (8.3) |  |
| 50k to 75k | 457 (11.0) | 17,927 (10.7) |  |
| 75k to 100k | 432 (10.3) | 13,276 (7.9) |  |
| 100k to 150k | 510 (12.2) | 15,582 (9.3) |  |
| 150k to 200k | 269 (6.4) | 7,063 (4.2) |  |
| More than 200k | 432 (10.3) | 8,892 (5.3) |  |
| Prefer Not To Answer | 613 (14.7) | 24,465 (14.5) |  |
| Skip | 206 (4.9) | 10,695 (6.4) |  |
